# Rapid Phenotypic and Genomic Change in Response to Therapeutic Pressure in Prostate Cancer Inferred by High Content Analysis of Single Circulating Tumor Cells

**DOI:** 10.1371/journal.pone.0101777

**Published:** 2014-08-01

**Authors:** Angel E. Dago, Asya Stepansky, Anders Carlsson, Madelyn Luttgen, Jude Kendall, Timour Baslan, Anand Kolatkar, Michael Wigler, Kelly Bethel, Mitchell E. Gross, James Hicks, Peter Kuhn

**Affiliations:** 1 Department of Cell and Molecular Biology, The Scripps Research Institute, La Jolla, California, United States of America; 2 Cold Spring Harbor Laboratory, Cold Spring Harbor, New York, United States of America; 3 Skyline Genomics, Roslyn Heights, New York, United States of America; 4 Department of Molecular and Cellular Biology, Stony Brook University, Stony Brook, New York, United States of America; 5 Department of Pathology, Scripps Clinic, La Jolla, California, United States of America; 6 Keck School of Medicine, University of Southern California, Los Angeles, California, United States of America; 7 Dornsife College of Letters, Arts and Sciences, University of Southern California, Los Angeles, California, United States of America; University of Nebraska Medical Center, United States of America

## Abstract

Timely characterization of a cancer's evolution is required to predict treatment efficacy and to detect resistance early. High content analysis of single Circulating Tumor Cells (CTCs) enables sequential characterization of genotypic, morphometric and protein expression alterations in real time over the course of cancer treatment. This concept was investigated in a patient with castrate-resistant prostate cancer progressing through both chemotherapy and targeted therapy. In this case study, we integrate across four timepoints 41 genome-wide copy number variation (CNV) profiles plus morphometric parameters and androgen receptor (AR) protein levels. Remarkably, little change was observed in response to standard chemotherapy, evidenced by the fact that a unique clone (A), exhibiting highly rearranged CNV profiles and AR+ phenotype was found circulating before and after treatment. However, clinical response and subsequent progression after targeted therapy was associated with the drastic depletion of clone A, followed by the sequential emergence of two distinct CTC sub-populations that differed in both AR genotype and expression phenotype. While AR- cells with flat or pseudo-diploid CNV profiles (clone B) were identified at the time of response, a new tumor lineage of AR+ cells (clone C) with CNV altered profiles was detected during relapse. We showed that clone C, despite phylogenetically related to clone A, possessed a unique set of somatic CNV alterations, including *MYC* amplification, an event linked to hormone escape. Interesting, we showed that both clones acquired *AR* gene amplification by deploying different evolutionary paths. Overall, these data demonstrate the timeframe of tumor evolution in response to therapy and provide a framework for the multi-scale analysis of fluid biopsies to quantify and monitor disease evolution in individual patients.

## Introduction

The androgen-androgen receptor (AR) signaling pathway is essential for the development and progression of prostate cancer and is a key target of many therapeutic agents [1]. In metastatic prostate cancer (PCa), androgen deprivation therapy (ADT), constitutes the gold standard treatment to induce tumor regression by suppressing AR activation. Despite initial response to ADT, patients often develop resistance and progress to castration resistant prostate cancer (CRPC), an incurable disease with poor prognosis. These patients are often treated with salvage hormone-directed therapies, including agents such as non-steroidal anti-androgens and androgen-synthesis inhibitors [1]. In managing these treatments, predicting therapeutic response and identifying early indicators of therapy resistance are major challenges. The levels of prostate specific antigen (PSA), an androgen regulated protein measured in the serum, is used to monitor therapeutic response in CRPC patients, however its predictive capability for this patient group is limited [2]. In addition, while many studies have identified molecular events that may contribute to therapeutic resistance to androgen-targeting agents, it is difficult to apply these findings due to the limited supply of sequentially acquired tissue and the expected heterogeneity across multiple metastatic deposits present in any individual patient [3], [4]. As such, methods that would allow for non-invasive sequential monitoring through the clinical course of therapy would be of tremendous value to clinicians.

Circulating tumor cells (CTCs) have the potential to provide a non-invasive means of assessing progressive cancers in real time during therapy, and further, to help direct therapy by monitoring phenotypic physiological and genetic changes that occur in response to therapy. In most CRPC patients, the primary tumor has been removed, and CTCs are expected to consist of cells shed from metastases, providing a ‘fluid biopsy’. Currently, the only method approved for CTC enumeration (CellSearch, Veridex) is based on an immune enrichment approach that pre-selects for cells that express Epithelial Cell Adhesion Molecule (EpCAM), an epithelial cell surface marker [5]. Although, numeric quantification of CTCs using CellSearch has yielded some prognostic information in certain cancers [6]–[8], this methodology has limitations such as low sensitivity (cells with low or absent EpCAM expression won't be captured) and the regular presence/contamination of genomically normal leukocytes in the sample preparation that hampers further molecular characterization and data interpretation. Recently, genomic changes based on array CGH and limited sequencing has been reported on CTCs isolated with the CellSearch system [9]. Detailed analysis in paired tumors and metastasis (n = 2) and CTCs (n = 8) suggested that most mutations detected in CTCs were present at a low-level in the primary tumor [9]. However, because a single timepoint during the clinical course of the disease was investigated this study does not address how a tumor may respond and evolve to therapeutic pressure.

Here, we demonstrate the power of two newly developed technologies in order to provide a more comprehensive portrait of the molecular changes occurring, at the single cell level, in a CRPC patient under the treatment pressure in both ADT and chemotherapy settings. The High Definition-CTC (HD-CTC) method was used for the longitudinal identification and enumeration of CTCs [10] and to asses for the expression of the AR [11]. The assay employs an unbiased protocol to examine and distinguish CTCs among the surrounding leukocytes based on their cytokeratin positive (CK+) phenotype by using a high resolution immunofluorescence imaging. In addition, the HD-CTC technology preserves the cell morphology in such a way that enables the morphometric and the indirect quantification of AR and CK protein expression levels for all the CTCs identified in the blood sample. To further characterize each CTC, a protocol was developed for extracting individual cells under conditions suitable for subsequent genomic analysis by a modification of the single nucleus sequencing method described by Navin, *et al.* [12] and Baslan, *et al.* [13]. The combined methods enabled to trace over time the molecular changes in the CTC population by correlating morphometric and protein expression data with genome wide CNV alterations for each of 41 individual CTCs isolated at four clinically significant timepoints. We were able to associate the emergence of distinct CTC subpopulations endowed with specific molecular alterations with the clinical course of the disease especially during the period of targeted ADT, which was clinically represented by a short period of response followed by resistance and clinical escape.

## Material and Methods

### Patient Clinical History and Blood Draws Collected During Treatment

The study was approved by the institutional review board (IRB) of University of Southern California Comprehensive Cancer Center. The patient provided written informed consent.

The patient presented with PCa metastatic to a lumbar vertebrae at diagnosis for which the primary biopsy represents the first specimen in this study. Initial treatment consisted of androgen deprivation therapy (leuprolide acetate). After 5 months, there was clinical progression to CRPC and the patient was enrolled in a clinical trial of docetaxel combined with bevacizumab and everolimus (clinicaltrials.gov identifier: NCT00574769). Before chemotherapy was initiated, a baseline blood draw was taken (Draw 1) according to the sample collection protocol. Clinical progression was noted after 4 months of protocol-specified chemotherapy. Over the next 3 months, additional doses of docetaxel as well as external-beam radiotherapy and samarium (^153^Sm) lexidronam (a bone-targeting radiopharmaceutical) were employed with limited palliative benefit. At 12 months after diagnosis, treatment with abiraterone acetate, a highly-selective androgen synthesis inhibitor, was initiated. Blood was drawn prior to starting abiraterone (Draw 2), at 3 weeks of continuous treatment coinciding with a clinical response represented by decreased pain and PSA level (Draw 3), and at 9 weeks coinciding with clinical progression represented by increasing pain and PSA levels (Draw 4). Following abiraterone, treatment was changed to cabazitaxel without clinical response followed by a rapid clinical deterioration. The patient died of widely metastatic prostate cancer 4 months following Draw 4 (17 months after diagnosis).

### Blood Sample Collection and Processing for CTC Detection

Patient peripheral blood samples were collected according to an IRB approved protocol. Samples were shipped to our laboratory and processed within 24 hours after the time of draw. Sample preparation was previously described in [10]. In brief, it consists of a red blood cell lysis followed by plating of the nucleated cells as a monolayer on custom made cell-adhesion glass slide followed by storage in a biorepository. Each sample produced at least 14 independent slides for CTC identification and characterization.

### Immunofluorescence Staining and CTC Enumeration

For this study, we used a protocol based on the published HD-CTC assay coupled with evaluation of androgen receptor (AR) status within the cytokeratin (CK) positive CTC population [11]. Briefly, the cells were labeled using mouse monoclonal cytokeratin 19 (1∶100; Dako) and panCK (1∶100; Sigma) primary antibodies to identify cytokeratin (CK) positive cells. AR positive HD-CTCs were identified using a rabbit anti-AR monoclonal antibody (1∶250, Cell Signaling Technology). Both the CK and AR antigens were visualized using AlexaFluor secondary antibodies; the CK primary antibodies were recognized with Alexa Fluor 555 IgG1 secondary antibody (1∶500, Invitrogen) and the rabbit AR antibody was recognized with Alexa Fluor 488 IgG (H+L) secondary antibody (1∶1000, Invitrogen). Alexa Fluor 647 conjugated anti-CD45 (1∶125; AbD Serotec) primary antibody was used to identify leukocytes as an exclusion marker. To confirm that the cells are nucleated and to enable the analysis of nuclear morphology all cells were stained with a 4',6-diamidino-2-phenylindole (DAPI).

The slides were imaged and putative CTCs were recorded using a computerized high-throughput fluorescence microscope at 10× magnification. CTCs were identified by a hematology technician using the previously published criteria of having a DAPI+ nucleus plus cytokeratin positivity and CD45 negativity [10]. Androgen receptor protein expression and localization were evaluated using two criteria (1) presence (AR^+^) or absence (AR^−^) of AR staining, and (2) AR subcellular localization (nuclear AR versus cytoplasmic staining or both). The threshold for AR positivity was defined as a signal more than 6 standard deviations over the mean signal intensity (SDOM) observed in the surroundings leukocytes (background). Subcellular localization was measured using the relative pixel density of AR staining over the nucleus and cytoplasm.

### HD-CTC Assay Reproducibility

The HD-CTC assay was technically validated with cell line spiking experiments to reach an R^2^ = 0.9997 on linearity testing as previously reported. These experiments were performed using SK-BR-3 cell lines and 0 to 3×10^2^ cells per mL of normal donor control blood. The coefficient of variation is 16% and inter-processor correlation is R^2^ = 0.979. Sample preparation process adhered to standard operating procedures for patient samples through a bar coded system for all consumables and instrumentation. All off-the-shelf instrumentation was calibrated according to the technical validation protocols established during the commissioning [14].

### Extraction of Single Cells

As a standard procedure, aimed at minimizing DNA fragmentation cells were picked within 5 days of the initial staining procedure. The experimental protocol for HD-CTC fluid phase capture was divided into three discrete sequential steps: (1) CTC relocation, (2) cell extraction and (3) isolation and manipulation of single CTCs for downstream molecular analyses.

HD-CTCs were relocated (step 1) using a transformation matrix from the initial data acquisition for HD-CTC identification. After calibration and relocation, each candidate cell was re-imaged at 40× resolution for the detailed morphometric analysis. For the cell extraction (step 2) an Eppendorf Transfer Man NK2 micromanipulator was used to capture the cell of interest inside a 25° jagged micropipette (Piezo Drill Tip ES, Eppendorf) by applying fluid suction. Once the cell of interest was captured inside the micropipette (step 3), the cell was rinsed with PBS and deposited inside a 0.2 mL PCR tube containing 2 µL of lysis buffer (200 mM KOH; 50 mM DTT). The sample was then and immediately frozen and stored at −80°C until further processing. All instruments and consumables were decontaminated using a DNAase solution and exposure to UV light for 30 min prior to the experiment.

### Single Cell Next Generation Sequencing and Bioinformatic Analysis

The cell containing vials were transferred in dry ice to the sequencing laboratory. Briefly, the lysed cell mixture was thawed and subjected to WGA and sequencing library construction as previously reported [13]. WGA was carried out manually in a 96-well plate format using the WGA4 Genomeplex Single Cell Whole Genome Amplification Kit (Sigma-Aldrich), followed by purification using a QIAquick 96 PCR Purification Kit (Qiagen). Concentration of eluted DNA was measured using a Nanodrop 8000 (Thermo Scientific). For each well, amplification was considered successful if the resulting DNA concentration was ≥70 ng/ µl (elution volume of 50 µl), followed by further Quality Control (QC) to confirm the appropriate sample size distribution using the Agilent 2100 Bioanalyzer (High-Sensitivity DNA Assay and Kit, Agilent Technologies).

In addition, detailed methods used to analyze sequencing data were published recently by our group [13]. Briefly, the informatics methods involves three steps: first, deconvoluting the sequence reads based on barcodes; second, mapping the reads to the human genome (hg19, Genome Reference Consortium GRCh37, UCSC Genome Browser database) [15], and removing PCR duplicates; and third, normalizing for guanine-cytosine (GC) content and estimating copy number using the CBS segmentation algorithm. The copy number profiles in this report are based on 20,000 variable length genome bins, averaging a length of ∼150 kilo-base pairs each, and were calculated as ratio compared to normal (hg 19). The data reported here had a median count of 1.78 million uniquely mapping reads, with a range from 244,190 (minimum cutoff 200,000) to 5.33 million.

### Cluster Analysis

The hierarchical clustering was performed in R [16] using the heatmap.2 function in the gplots package. Ward's method with Euclidean distance metric was used for the clustering. The heatmap is colored according to the cutoffs described above and the clustering was performed using median centered data.

### Frequency Analysis to Define Genomic Alterations

Using median centered CNV profiles, cutoff ratios versus the median of 0.8 and 1.25 were used to define deletions and amplifications, respectively. These cutoffs were used both to color the heatmap and to do the frequency analysis.

### Statistics and Cell Morphology Analysis

The cell shape (*cell roundness*) was analyzed by tracing the cell cytoplasm contour in the composite image of each CTC. The traced cell image was imported into R, and an ellipsis was fitted to the shape using a least squares fitting algorithm described by Halir and Flusser [17]. The algorithm outputs the cell's major axis, which is the largest radius of the fitted ellipsis (Refer to supporting information). The cell roundness (*c*) is estimated as the fraction of the *de facto* cell area (*A*) and the area of a circle with the radius (*r*) set to the cell's major axis.
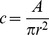



The p-value used in the comparison of the roundness between the CTCs in Draw 3 and 4 was calculated using the Wilcoxon sum-rank test.

## Results and Discussion

### Treatment Response Monitored by Longitudinal CTC Molecular Analysis

**Figure 1:**
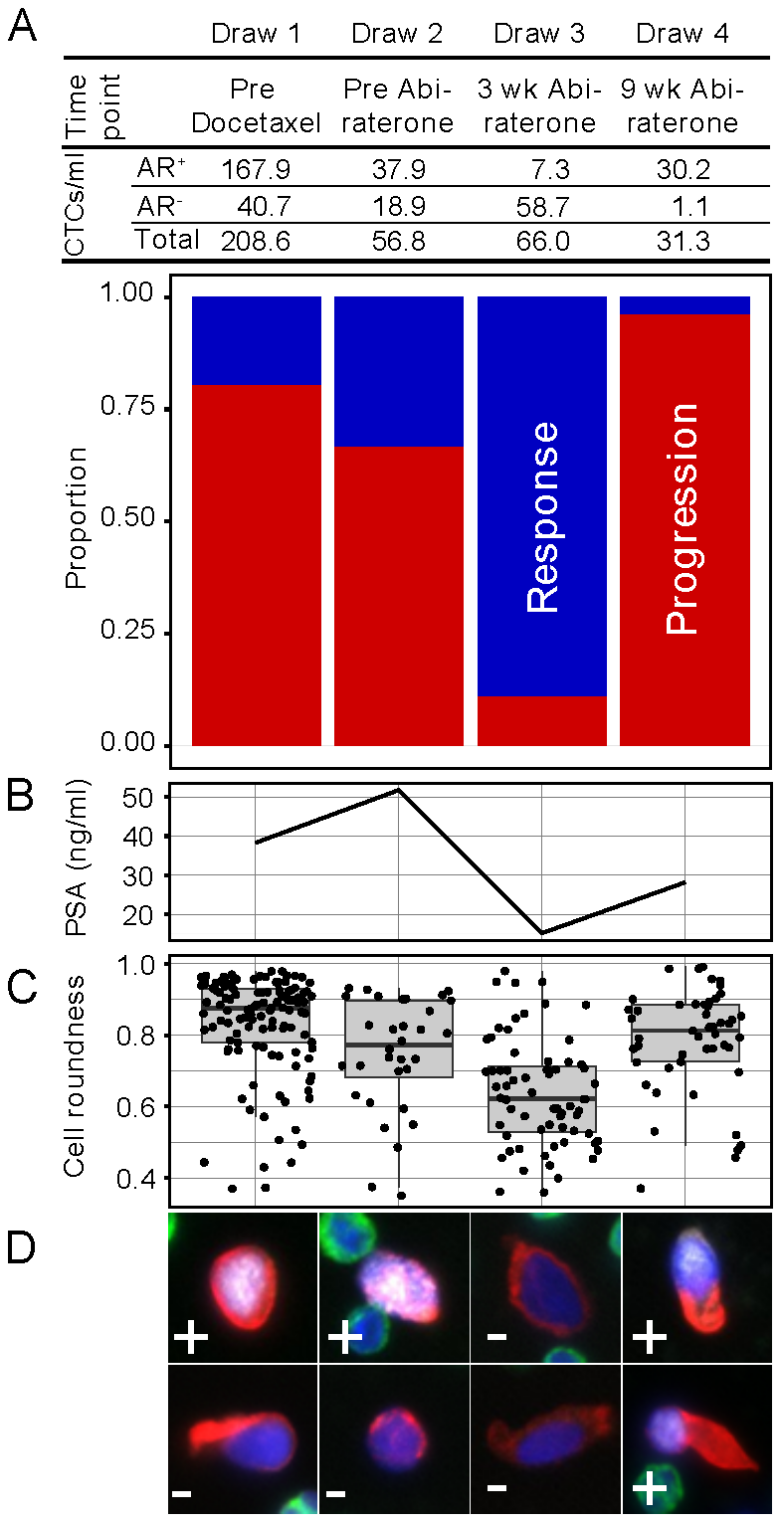
Abiraterone acetate induces phenotypic alterations in the CTC population. (A) The total HD-CTCs counts, including the number of phenotypically distinct AR^+^ and AR^−^ cells, was determined for each blood Draw collected during therapeutic intervention. CTCs were defined as AR positive if the AR signal intensity was higher than six standard deviations over the mean (SDOM) of the surrounding leukocytes (background). The bar-graph shows the change in the distribution of the AR^+^ and AR^−^ CTC subpopulations along the course of treatment, indicated in red and blue respectively, and the numbers are presented above each bar. (B) PSA concentration measured at each treatment timepoint. (C) Boxplot of cell roundness for each individual CTC identified across the different treatment timepoints. (D) Representative 40× immunofluorescence images of AR^+^ and AR^−^ HD-CTCs from the subpopulations identified in each treatment timepoint. Immunofluorescence channels are colored as follows: nucleus: blue; cytokeratin: red; AR: white; and CD45: green. AR phenotype is indicated in the bottom left corner of each image. All graphs were constructed using the ggplot2 and rgl packages in R.

In order to assess the patient's response to treatment high content single cell analysis including: (1) AR protein expression phenotype, (2) AR subcellular localization and (3) CNV genomic profiling were performed in the CTCs identified in the blood samples collected across four different intervals representing decision points in the standard care of CRPC including: (Draw 1) immediately prior to initiation of docetaxel based chemotherapy, (Draw 2) immediately prior to abiraterone acetate (a highly-selective androgen synthesis inhibitor), (Draw 3) after three weeks, and (Draw 4) after nine weeks of continuous abiraterone treatment. The specific data for all profiled cells is presented in the supporting information. In addition, a similar sequencing based method was used to obtain the CNV profile of one metastatic site from the patient using a bone biopsy taken at the time of diagnosis (5 months prior to draw 1) prior to receiving any cancer-specific therapy. As shown in Figure 1A, and during the 7 month period between Draws 1 and 2, the patient exhibited initial response to docetaxel-based chemotherapy followed by resistance. Concurrently, the patient's fluid biopsy showed a constant proportion of AR^+^ and AR^−^ subpopulations while the overall number of CTCs declined (Figure 1A, 1D, Figure S1 and Table S1).

**Figure 2:**
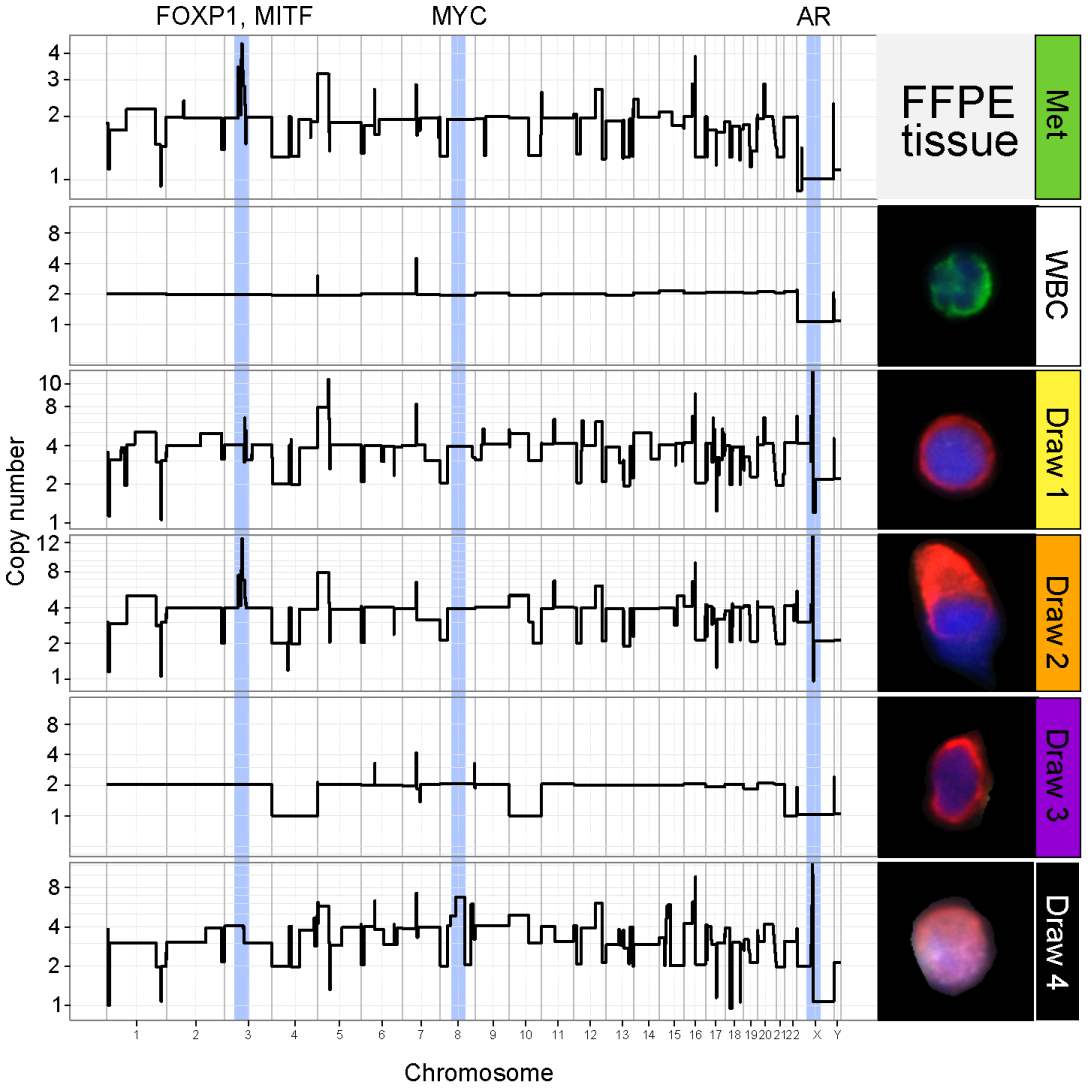
Concurrent phenotypic and genotypic profiling of single prostate tumor cells. Copy number variation profiles from the patient's bone metastasis; a control single WBC; and single CTCs from each of the four treatment timepoints are shown. The corresponding fluorescent image of the cell used to generate the CNV profile is shown to the right. Relevant genomic alterations and their chromosome localizations occurring in each specific draw are indicated with pale blue bars.

**Figure 3:**
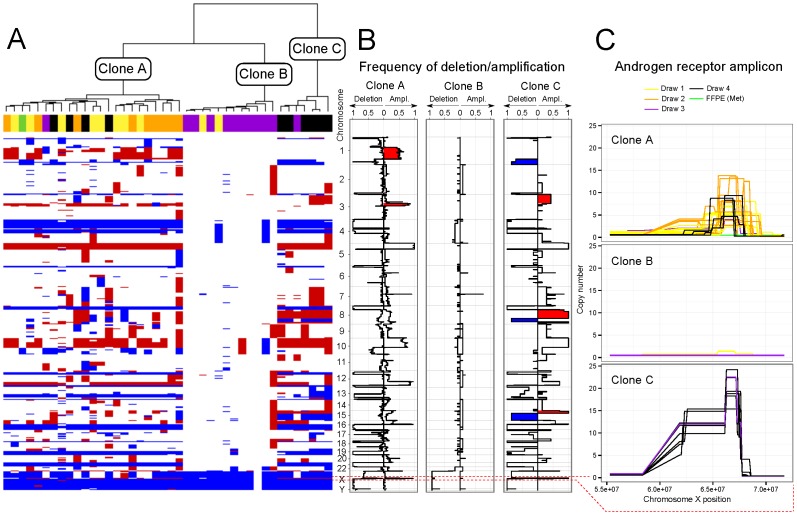
Clonality and genomic aberrations in the CTC population. (A) Three different clonal lineages, represented as Cluster A, B and C, were identified based on the comparison of 41 single cell CNV profiles in an unsupervised hierarchical clustering. The blood draw from which each cell was isolated is indicated as Draw 1: yellow; Draw 2: orange; Draw 3: purple; and Draw 4: black. For reference, the bone metastasis FFPE tissue was included in the tree, colored in green. Below the tree, a heatmap indicates the amplifications (red) and deletions (blue) across the entire genome of each individual cell. (B) Frequency of genomic amplifications and deletions in the three clusters identified. Areas uniquely amplified (red) or deleted (blue) in cluster A and C are highlighted. (C) A detail plot of the AR amplification event colored per draw for each individual cluster is shown.

The genomic CNV profiles of CK^+^ cells from Draws 1 and 2 were of two types (Figures 2 and S2). Three of these cells were negative for AR expression (CK^+^AR^−^) while the majority (16/19) showed high levels of AR protein (CK^+^AR^+^). One AR^−^ and one AR^+^ cell had near normal CNV profiles comparable to those obtained from single CK^−^CD45^+^ leukocytes (Figure 2). All other CK^+^AR^+^ cells exhibited a complex pattern of genomic rearrangements that were similar to the genomic profile obtained retrospectively from the patient's bone metastasis (hormone naïve tissue sample) obtained at diagnosis (Figure 2 and Figure 3A). The CK+AR+ cells and the bone metastasis sample shared multiple gains and losses of chromosome arms plus a characteristic focal amplification on 3p13 centered on the phosphatase regulatory subunit PPP4R2 and containing at least two genes implicated in cancer, FoxP1 [18], [19] and MITF [20] (Figure 2). To the level of resolution available, each of the shared events showed identical genomic breakpoints, and in the hierarchical clustering analysis the AR^+^ cells from draws 1 and 2 clustered together with the bone metastasis (Cluster A in Figure 3A). From this evidence, we infer that these cells are *bona fide* CTCs derived from the patient's metastatic lineage. Despite the clear lineage relationship, the AR^+^ circulating cells differed from the metastasis at the AR locus, showing multicopy amplification of various segments on Xq12 containing the AR gene itself. AR amplification is frequent in CRPC, and has been linked to progression from castration-sensitive prostate cancer to CRPC [21]. It is noteworthy that each of the AR amplifications (Figure 3C) are unique, arising from multiple different breakpoints on either side of the AR gene, indicating that AR amplification arose multiple independent times (convergent evolution) likely as result of the selective pressure imposed by the androgen deprivation therapy.

At Draw 3, after three weeks of abiraterone acetate treatment, the patient displayed a clear clinical response as defined by decrease in PSA and pain (Figure 1B). This response coincided with an abrupt change in CTC phenotypes and genotypes. Although the absolute number of CTCs in Draw 3 was comparable to that of Draw 2, there was an almost complete depletion of the AR^+^ CTC population (Figure 1A). The CK^+^ cells identified in Draw 3 expressed little or no AR protein and also differed morphologically, appearing to be significantly more elongated than the AR^+^ cells from Draws 1 and 2 (Figure S1 and Table S1). This morphological change is reflected in a decrease in the median cell roundness (Figure S3) from 0.87 (sd = 0.14) in Draw 1 and 2 to 0.62 (sd = 0.15) in Draw 3, p<10^−11^ Wilcoxon rank-sum test (Figure 1C).

The apparent effect of treatment was also evident in the genomic analysis of Draw 3 where the altered phenotypic states correlated with distinct genomic profiles. The majority (10/12) of phenotypically AR^−^ cells from Draw 3 were not amplified for AR and exhibited apparently normal or near normal (pseudodiploid) profiles (Figure S2) placing them in Cluster B in Figures 3A. One of the two AR^−^ cells from this timepoint had the CNV signature typical of Cluster A including amplification of AR, while the other associated with a third cluster (Cluster C in Figure 3A), dominated by cells from the subsequent timepoint (Draw 4). Missense mutations affecting AR protein stability and/or nonsense mutations in the AR gene could account for the AR phenotype-genotype disparity in the last two cells. We interpret that the initial response to abiraterone acetate significantly depleted the androgen-dependent AR^+^ population, and that another AR^−^ population dominated by pseudodiploid cells was present in the circulation. Based on the total cell count, staying constant between draws 2 and 3, we infer that the Draw 3 population is a consequence of cancer, but from a source outside of the main tumor lineage (Figure 3A).

Draw 4 was collected at the point of clinical progression, when PSA levels increased after 9 weeks on abiraterone (Figure 1B). At this point, the CTC count had decreased to 47% of the previous timepoint, but had once again undergone a significant phenotypic shift, as the majority of CTCs were once again AR^+^ with a cell roundness value of 0.81 typical of cells from the first two draws (Figure 1C and Figure S1). This finding, suggesting an association between therapy response and a CTC phenotype rather than with total CTC count, is consistent with a recently published study where the expression of two markers for the AR signaling pathway on CTCs was monitored in response to androgen-directed therapy [22].

Alterations in response to therapy were again apparent at the genomic level, as (6/10) cells formed the majority of a new, apparently clonal, subpopulation (Cluster C in Figures 3A and S2). The CNV signatures in Cluster C are clearly in the original lineage, going back to the bone metastasis sampled before any systemic therapy, but is now characterized by functionally relevant events such as a narrow amplicon containing MYC, and the disappearance of the FOXP1/MITF amplicon along with other differences noted in Figures 2, 3A and 3B. MYC amplification is one of the most common alterations observed in metastatic tumors, and has been suggested to be a bypass mechanism for AR independent resistance [23]. Interestingly a closer examination of the genomic AR amplification (outlined in Figure 3C) shows that, in contrast to the heterogeneous amplification boundaries observed in earlier cells (cluster A), the cells in cluster C exhibit a single profile shape with nearly uniform breakpoints and significantly higher levels of AR amplification. Taken together the genomic elements suggest that the Cluster C cells represent a novel lineage, apparently resistant to abiraterone acetate, and generated perhaps from a single resistant cell.

**Figure 4:**
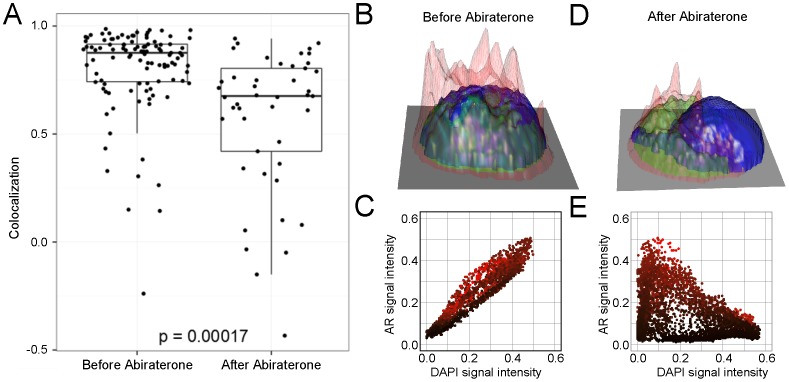
AR subcellular localization changes at the time of disease progression. (A) Comparison of the AR subcellular localization in the CTCs identified in the blood prior to and after nine weeks of abiraterone treatment. Correlation between the AR and DAPI signals within the cell is indicative of AR being colocalized with DAPI, *i.e.* localized in the cell nucleus. High correlation was generally seen before abiraterone treatment, but a shift to less nuclear stain was observed after nine weeks of treatment (p = 0.00017, Wilcoxon sum-rank test). (B) and (D) Height maps constructed from the pixel intensities of CK (red), AR (green) and DAPI (blue) in representative CTCs to visualize the subcellular localization of AR. The cell in (B) was isolated before abiraterone initiation and displays AR staining confined to the nucleus, while cytoplasmic AR staining is observed in the CTC identified at the time of therapeutic relapse (D). (C) and (E) Plots of AR versus DAPI signal intensities for each pixel inside the cell in the 40× images of the CTCs in (B) and (D), respectively. Each plot point is colored by the corresponding CK signal intensity. Nuclear localization was observed as positive correlation between the two intensities (C), and nuclear exclusion as negative correlation (E). All graphs and were done using the ggplot2 and rgl packages in R.

In addition, morphometric analysis of AR subcellular localization showed that AR was generally localized in the nucleus of cells from Draws 1 and 2, but was identified as significantly less localized to the nucleus in the CTCs isolated in Draw 4 collected at progression (p = 0.00017 Wilcoxon rank-sum test) (Figure 4). This finding is particularly interesting in the light of recent studies indicating that ligand independent AR splice variants may mediate abiraterone resistance in a human CRPC xenograft model [24], and that these truncated and constitutively active forms of AR is found to be localized in the nucleus as well as cytoplasm in prostate cancer cell lines [25].

Although our study is based on longitudinal study of a single patient, our findings are consistent with previous studies involving genomic analysis from either CTCs or circulating cell-free DNA isolated from patients with metastatic prostate cancer [26], [27]. However, these prior studies were generally limited to the characterization of pooled samples from a single timepoint, and therefore do not shed light into the temporal and dynamic evolution of cancer under therapeutic selective pressure. Regardless, consistent with these prior reports we observed copy number alterations in chromosome 8 (particularly gain in 8q and loss in 8p), which is one of the most frequent somatic mutations described in prostate cancer [18]. In addition, our finding that AR amplification was not found in sample obtained before initial androgen-deprivation therapy, but occurred at high frequency in later samples representing CRPC is consistent with multiple prior studies linking AR amplification with androgen-independent prostate cancer growth.

### A Time Course of Tumor Evolution

Clonal evolution of cancer is a well-established principle that has been validated in multiple published studies [28]–[30], as well as, the appearance of somatic mutations in tumors in response to therapeutic selective pressure [31], [32]. We interpret the phenotypic and genotypic changes in circulating cell populations presented here as representing sequential steps of genetic evolution in response to a multi-step therapeutic regime culminating in treatment with abiraterone acetate.

The bulk metastatic biopsy taken prior to initiation of therapy provides the root CNV profile to from which the subsequent time course CTC profiles have evolved. It exhibits a backbone of CNV elements that defines a lineage, based on CNV breakpoints, that is carried forward in the circulating cells from blood draws taken during later treatment. The first two of these draws were taken after an initial course of androgen deprivation therapy (ADT) (leuprolide acetate). One population in Draws 1 and 2 (Clone A) was a clearly a direct descendant of the met biopsy profile with the exception that all cells showed high-copy AR amplification and strong AR protein expression. We interpret these cells to be the products of metastatic deposits that had evolved to amplify the AR gene locus and overexpress androgen receptor protein as a result of genetic selection for resistance to the initial round of ADT. It is noteworthy that in addition to the AR amplified cells, both draws contained a significant fraction of cytokeratin positive, AR negative cells with near-normal (pseudodiploid) genomes forming a separate CNV cluster (Figure 3). It is also interesting that the clonal structure of the AR+ cells changed very little between Draws 1 and 2 despite intervening rounds of chemotherapy and radiation therapy over a period of 7 months.

In contrast to the similarity of cell phenotype and genotype in Draws 1 and 2, the selective effects of abiraterone acetate were very evident in Draws 3 and 4. After three weeks of treatment the androgen dependent, AR positive cells in Draws 1 and 2 were nearly absent and the CK+ population consisted almost entirely of AR negative pseudodiploid cells. The clone (Clone C) that would become dominant at the nine-week timepoint (Draw 4) was first seen as a single incidence in Draw 3. By Draw 4, AR+ cells had once again become a substantial fraction of the population, albeit with a significantly altered CNV profile (Figure 3). We thus infer that Clone C was selected as a drug-resistant subclone from one of the initially depleted metastatic sites. That the early and late stage clones are clearly related and stem from the same lineage is evident from the frequency graphs in Figure 3B, showing that most events are maintained and have identical boundaries. Several other events, however, are either new, deletions on 1q, 8q, and 15q and gains of 3p, 15p, and complex rearrangement of 8q involving a separate amplification of a narrow region containing MYC, or are more frequent in the late stage cells. The co-occurrence of MYC amplification along with re-emergence of AR protein expression and AR amplification may have important therapeutic implications as c-Myc expression confers androgen-independent growth [23]. While c-Myc has proved a difficult therapeutic target, strategies which target key metabolic and other changes downstream of c-Myc activation are being investigated in many clinical trials [33]. Our data suggests that co-targeting of c-Myc along with AR may provide an approach to delay or prevent the emergence of resistance to abiraterone acetate and other androgen-targeting agents.

Through this selective process, the population of AR negative, pseudodiploid cells remained a significant fraction of cytokeratin positive cells. The presence of these phenotypically (Figure 1A) and genotypically (Figure 3A) distinct cytokeratin positive cells raises the question of their origin. Previous studies have consistently identified cells in primary tumor tissue with similarly unaltered or pseudodiploid CNV profiles [12]. We also cannot exclude that they represent a pre-existing minor population of normal epithelial cells exposed by depletion of the cancer cells in Draw 3, however, that the number of these cells in Draw 3 was comparable to the numbers in Draws 2 and 4 would make that less likely. Alternatively, they may represent tumor associated macrophage lineage cells with phagocytosed intracytoplasmic cytokeratin sloughed off from tumor sites as they are depleted of sensitive cells or a castration resistant stem-like tumor cell population recently described in engrafted prostate tumors and phenotypically characterized as CK^+^ AR^−^ cells [34]. However, further interrogation of single point mutations combined with protein expression analysis will be required to gain insight into the nature of these cells and their role in tumor progression, if any.

In an era of clinical oncology that is progressively moving towards targeted cancer therapy, approaches that allow for non-invasive monitoring of therapeutic response at both phenotypic and genetic levels are essential. We have chosen to approach this goal through a combined phenotypic and genetic analysis of non-leukocyte circulating nucleated cells, without a pre-selection step that may bias the CTC population. Our method allows us to correlate genomic events with complex phenotypes based on protein expression and cell morphology. Alternative methods, such as sequencing of free DNA from plasma (ctDNA) are also powerful tools and can yield both mutation and copy number information, but only for an admixture of the various cellular components [35]. In this case study, we show the remarkable extent and speed of the genomic reorganization as putative-resistant clones emerge at the time of treatment failure. Although, we cannot establish a mechanistic relationship between the large CNV changes and the eventual resistance to abiraterone acetate based on a single patient, it appeared that after 9 weeks of targeted therapy the original CTC population was not completely eliminated and an apparently drug-resistant clone was present. Finally, the integration of data across multiple subjects will open the door for a deeper understanding of the mechanisms and timing of resistance and allow for rationally-designed, personalized treatments based on sequential, combined, or intermittent application of therapeutic agents.

## Supporting Information

Figure S1 **Representative gallery of 40× high resolution immunofluorescence images of the two phenotypically distinct CTCs subpopulations identified.** A and B, Composite and non-merged images of an AR+ and AR− HD-CTC isolated from pre docetaxel (A) and pre abiraterone (B) treatment timepoints. C and D, Two different AR− and AR+ HD-CTCs, the predominant tumor cell phenotypes found in 3 (C) and 9 weeks post abiraterone (D). Panel D, CTCs with different pattern of AR subcellular localization. Nuclear and cytoplasmic AR is shown in the top panel and nuclear AR in the bottom panel. Composite and non-merged images for the individual immunofluorescence channels were colored as followed: DAPI (blue); cytokeratin-CK (red), androgen receptor-AR (white) and CD45 (green).
(DOCX)

Figure S2 **Complete collection of single CTC CNV profiles.** The genome wide copy number fingerprints for all successfully profiled cells at each of different treatment timepoint.
(DOCX)

Figure S3 **Examples of cell roundness estimation.** The cell shape was analyzed by tracing the cell cytoplasm contour in the composite image of each CTC. The traced cell image was imported into R, and an ellipsis was fitted to the shape using a least squares fitting algorithm described by Halir and Flusser. Black line represents the manually drawn cell outline, red line the fitted ellipse. The cell roundness is estimated as the fraction of the *de facto* cell area and the area of a circle with the radius set to the cell's major axis. The cell roundness calculated to be 0.62 for the oval-shaped cell (left) and 0.96 for the more rounded cell (right). The p-value used in the comparison of the roundness between the CTCs isolated between the different draws was calculated using the Wilcoxon rank-sum test.
(DOCX)

Table S1 **Summary of the different phenotypic and genotypic traits analyzed in the 41 individual cells profiled for copy number alterations.** Concordance between AR phenotype-genotype was determined by comparison of the AR amplification status with the AR staining phenotype (Negative or Positive) for each individual cell. In red are cells that exhibited discordant AR phenotype-genotype.
(DOCX)
