# Supplementary material for: Rapid Phenotypic and Genomic Change in Response to Therapeutic Pressure in Prostate Cancer Inferred by High Content Analysis of Single Circulating Tumor Cells
Source: PLoS One. 2014 Aug 1;9(8):e101777. doi: 10.1371/journal.pone.0101777 (PMC4118839; doi:10.1371/journal.pone.0101777)
Supplement: Figure S1 — Representative gallery of 40× high resolution immunofluorescence images of the two phenotypically distinct CTCs subpopulations identified. A and B, Composite and non-merged images of an AR+ and AR− HD-CTC isolated from pre docetaxel (A) and pre abiraterone (B) treatment timepoints. C and D, Two different AR− and AR+ HD-CTCs, the predominant tumor cell phenotypes found in 3 (C) and 9 weeks post abiraterone (D). Panel D, CTCs with different pattern of AR subcellular localization. Nuclear and cytoplasmic AR is shown in the top panel and nuclear AR in the bottom panel. Composite and non-merged images for the individual immunofluorescence channels were colored as followed: DAPI (blue); cytokeratin-CK (red), androgen receptor-AR (white) and CD45 (green). (DOCX) [file pone.0101777.s001.docx]

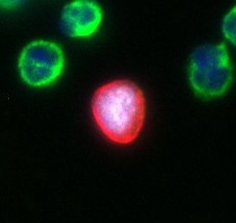

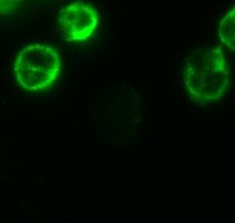

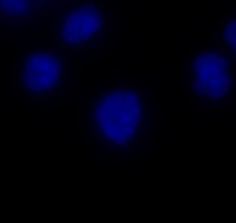

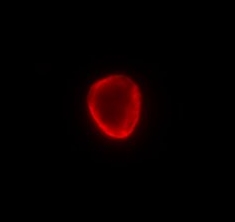

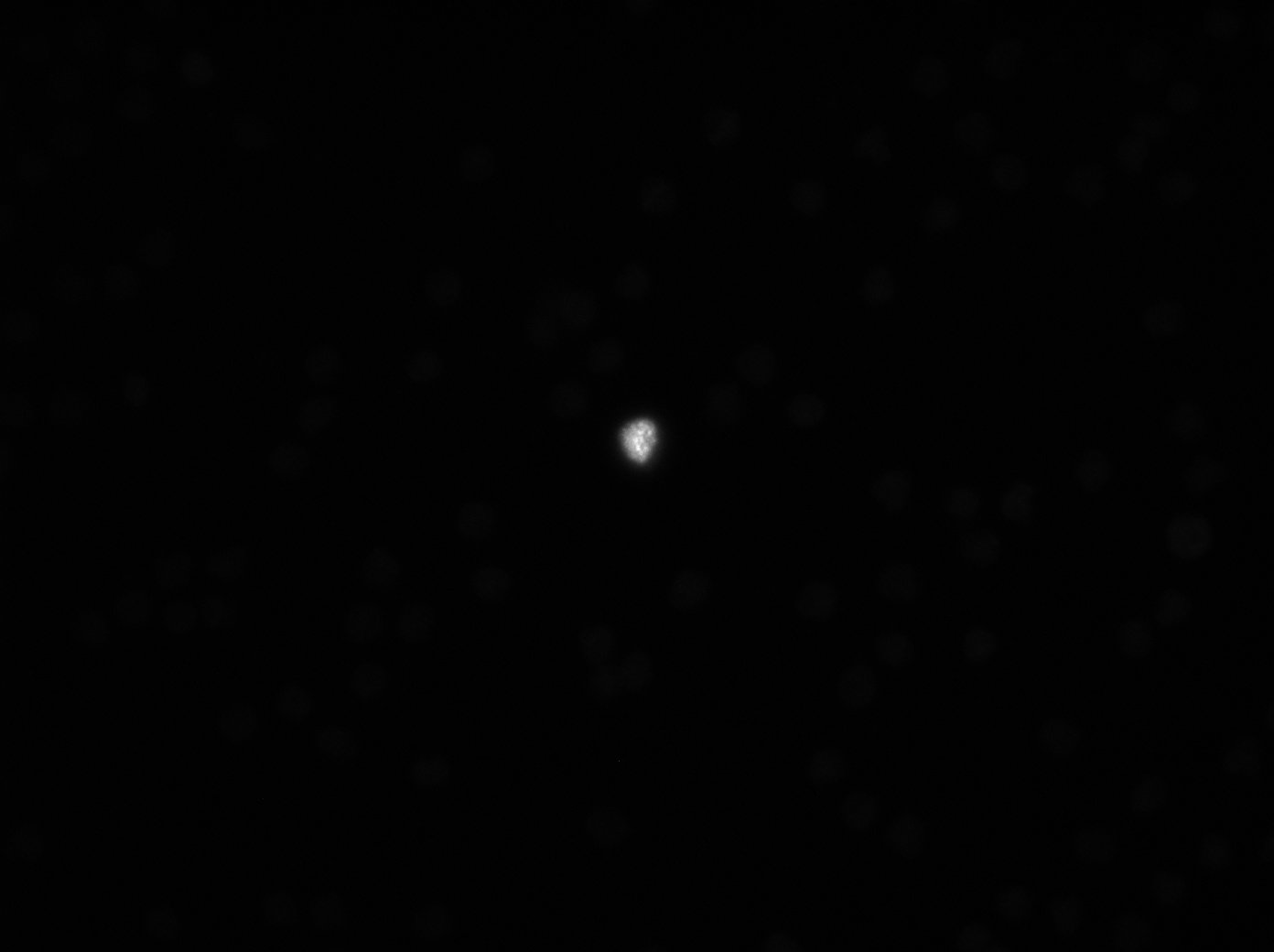

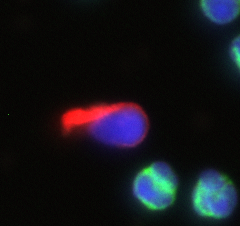

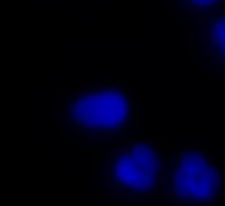

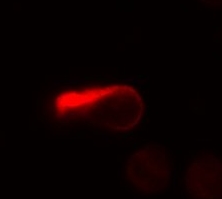

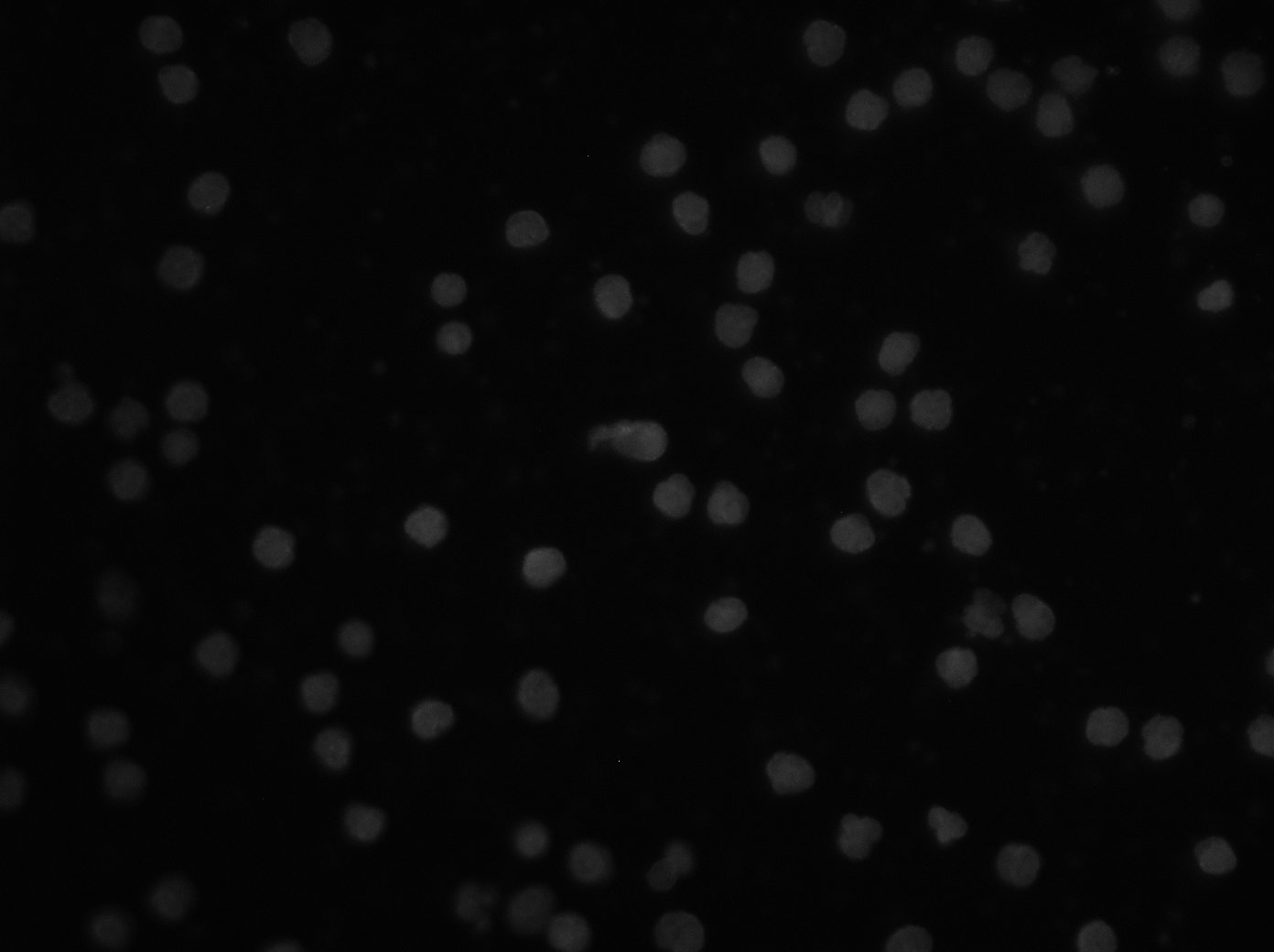

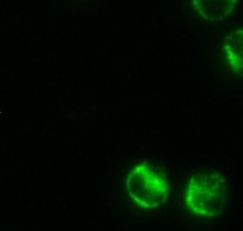


**A) Pre-Docetaxel/Bevacizumab-Draw 1**

HD-CTC

AR+

HD-CTC

AR-

DAPI

CK

AR

CD45

Composite

**B) Pre Abiraterone acetate-Draw 2**

HD-CTC

AR+

HD-CTC

AR-


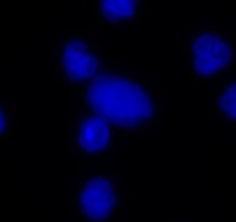

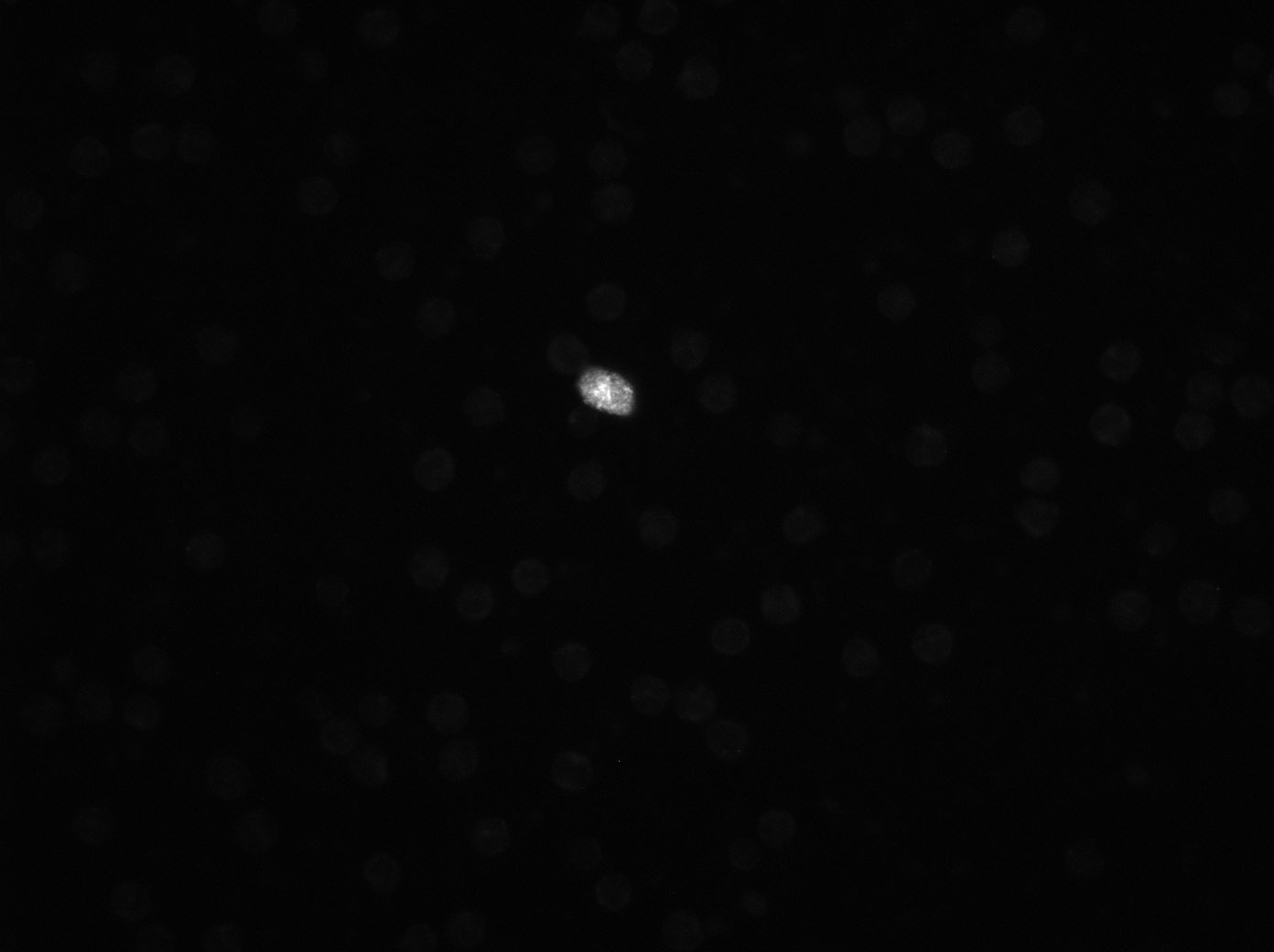

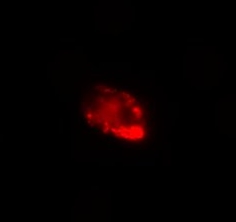

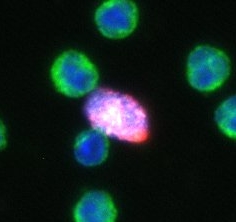

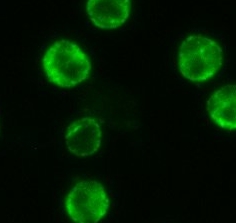

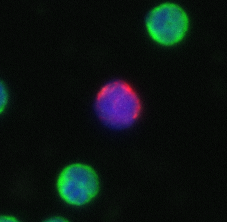

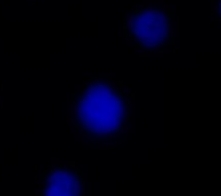

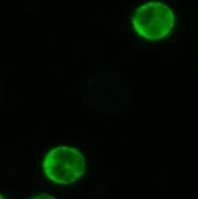

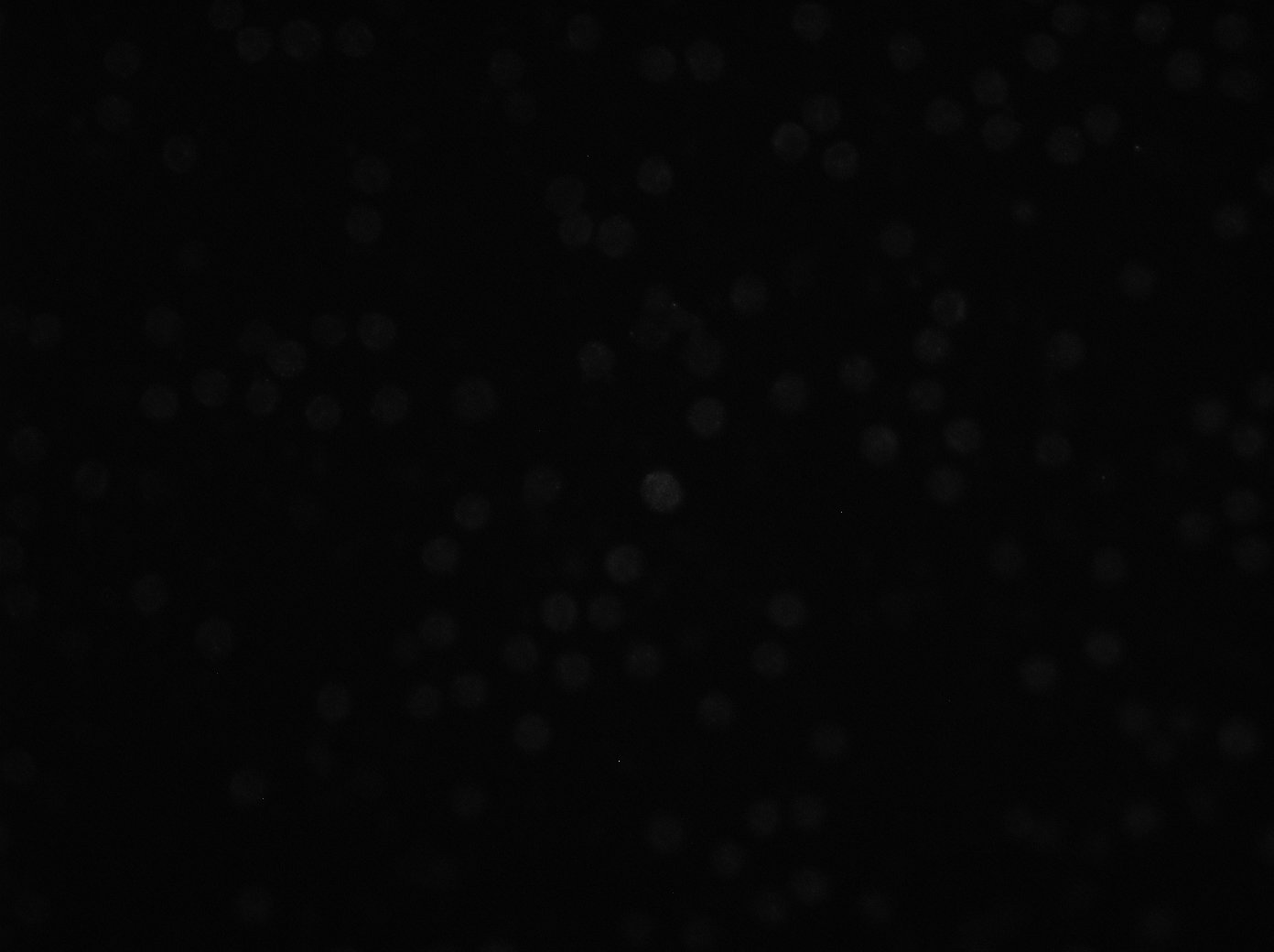

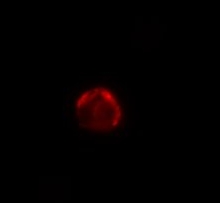


DAPI

CK

AR

CD45

Composite

**C) Three weeks Post Abiraterone acetate- Draw 3**


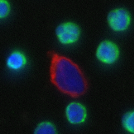

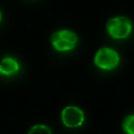

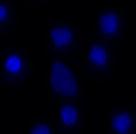

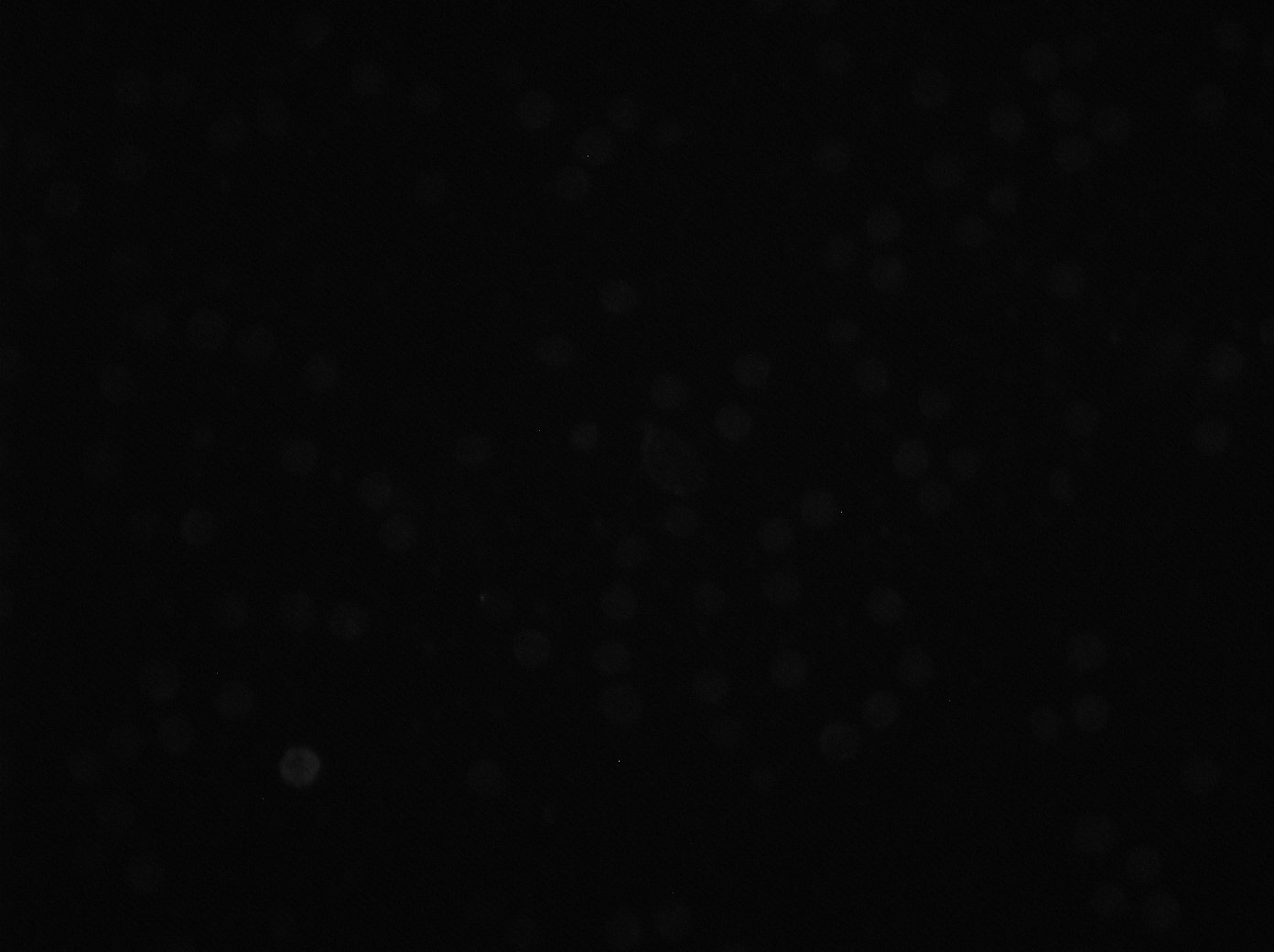

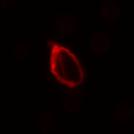

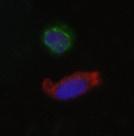

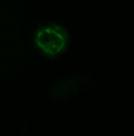

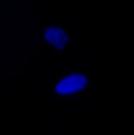

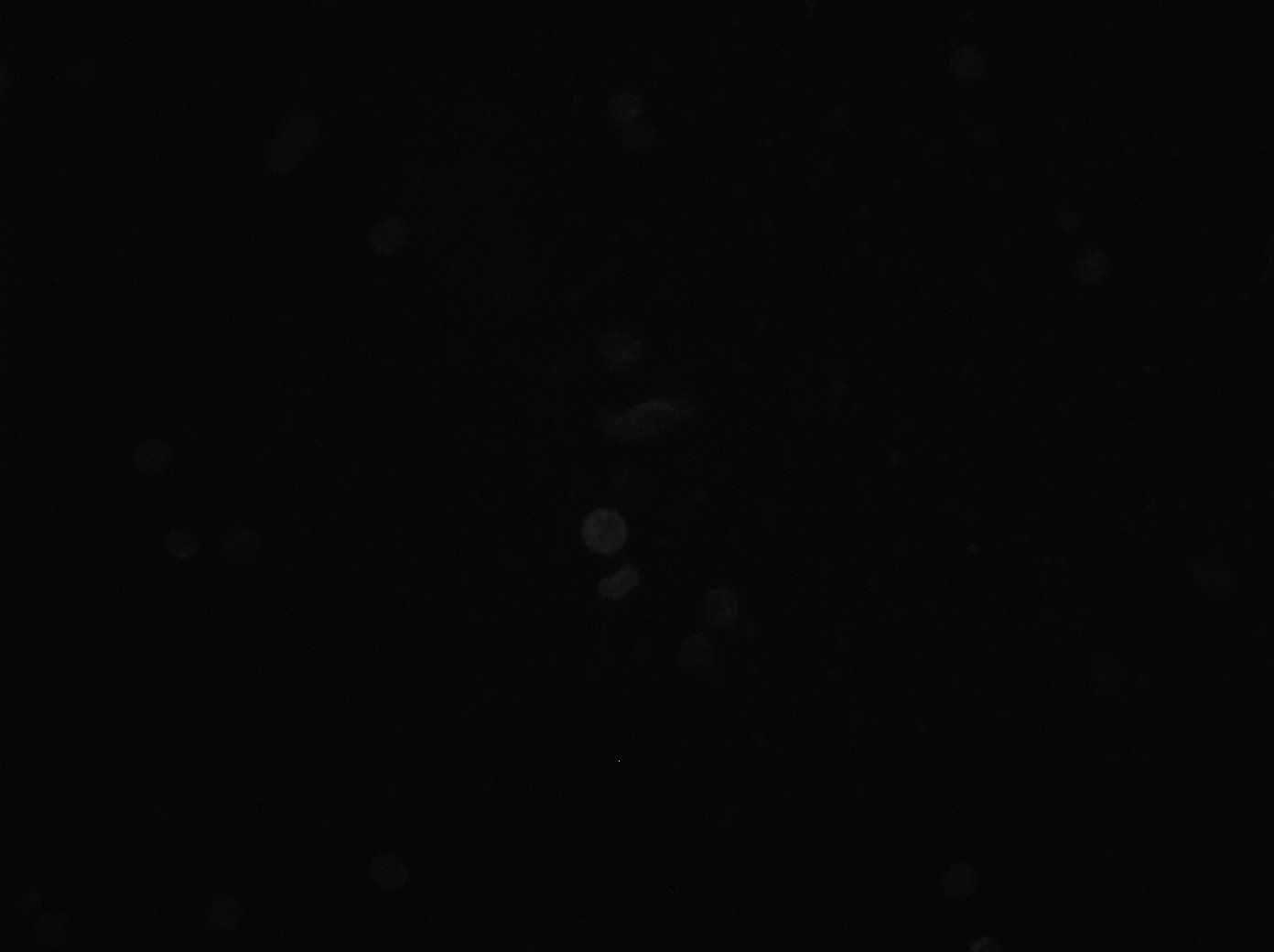

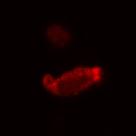


HD-CTC

AR-

HD-CTC

AR-

DAPI

CK

AR

CD45

Composite

**D) Nine weeks Post Abiraterone acetate-Draw 4**


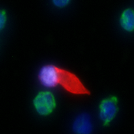

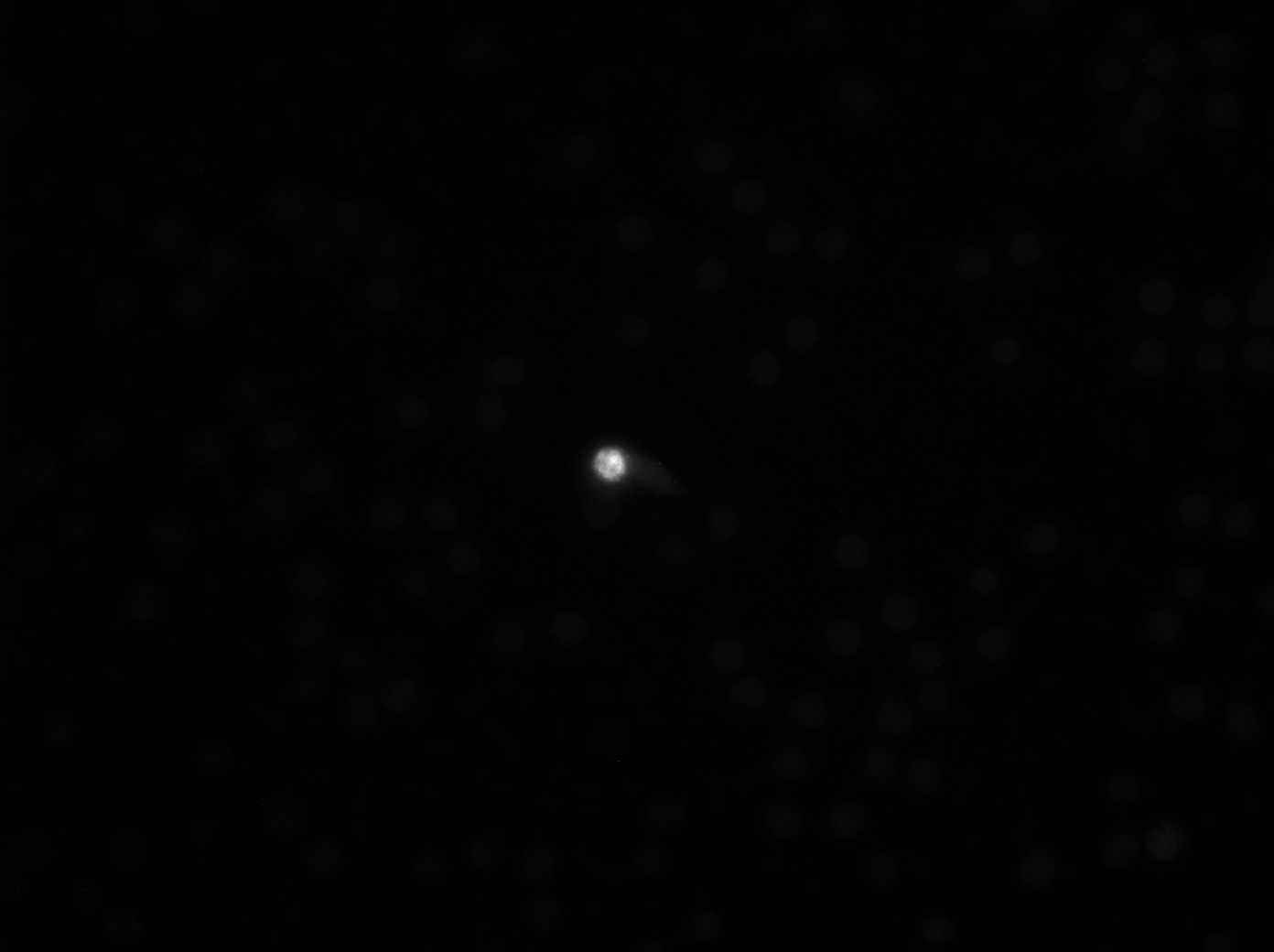

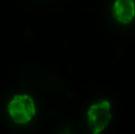

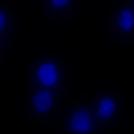

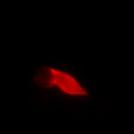

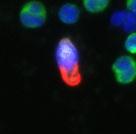

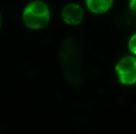

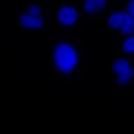

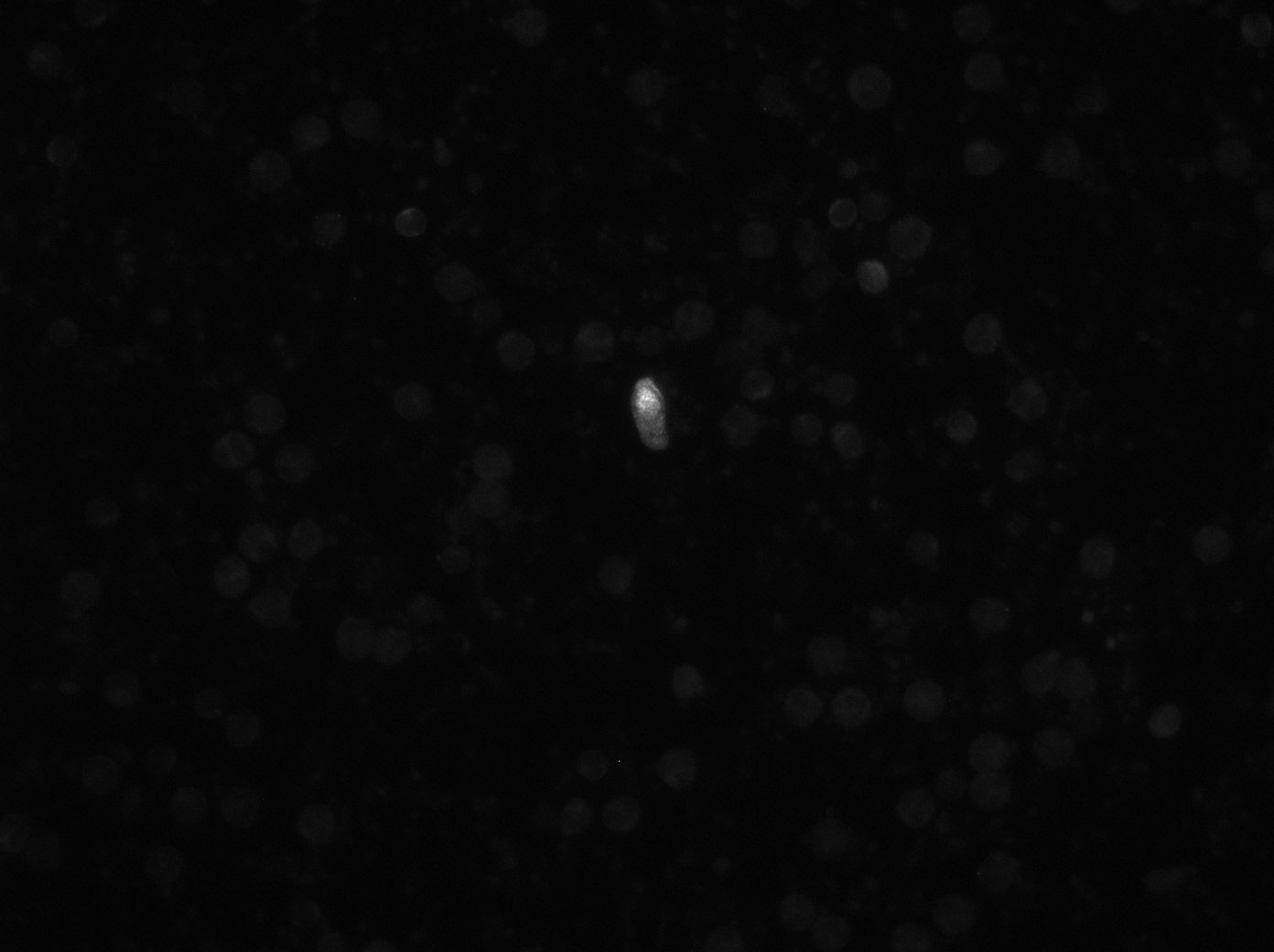

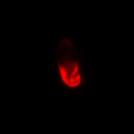


HD-CTC

AR+

HD-CTC

AR+

DAPI

CK

AR

CD45

Composite

**Figure S1. Representative gallery of 40X high resolution immunofluorescence images of the two phenotypically distinct CTCs subpopulations.** (A) and (B) Composite and non-merged images of an AR+ and AR− HD-CTC isolated from pre docetaxel (A) and pre abiraterone (B) treatment timepoints, are depicted. (C) and (D) Two different AR− and AR+ HD-CTCs, the predominant tumor cell phenotypes found in 3 (C) and 9 weeks post abiraterone (D) are shown. In panel (D) CTCs with different pattern of AR subcellular localization are depicted. Nuclear and cytoplasmic AR is shown in the top panel and nuclear AR in the bottom panel. The composite and non-merged images for the individual immunofluorescence channels were colored as followed: DAPI (blue); cytokeratin-CK (red), androgen receptor-AR (white) and CD45 (green).
