# Supplementary material for: Rapid Phenotypic and Genomic Change in Response to Therapeutic Pressure in Prostate Cancer Inferred by High Content Analysis of Single Circulating Tumor Cells
Source: PLoS One. 2014 Aug 1;9(8):e101777. doi: 10.1371/journal.pone.0101777 (PMC4118839; doi:10.1371/journal.pone.0101777)
Supplement: Figure S2 — Complete collection of single CTC CNV profiles. The genome wide copy number fingerprints for all successfully profiled cells at each of different treatment timepoint. (DOCX) [file pone.0101777.s002.docx]

**Figure S2.**

**Figure S2. Complete collection of single CTC CNV profiles.** The genome wide copy number fingerprints for all successfully profiled cells at each of different treatment timepoint are shown.
