# Supplementary material for: Rapid Phenotypic and Genomic Change in Response to Therapeutic Pressure in Prostate Cancer Inferred by High Content Analysis of Single Circulating Tumor Cells
Source: PLoS One. 2014 Aug 1;9(8):e101777. doi: 10.1371/journal.pone.0101777 (PMC4118839; doi:10.1371/journal.pone.0101777)
Supplement: Figure S3 — Examples of cell roundness estimation. The cell shape was analyzed by tracing the cell cytoplasm contour in the composite image of each CTC. The traced cell image was imported into R, and an ellipsis was fitted to the shape using a least squares fitting algorithm described by Halir and Flusser. Black line represents the manually drawn cell outline, red line the fitted ellipse. The cell roundness is estimated as the fraction of the de facto cell area and the area of a circle with the radius set to the cell's major axis. The cell roundness calculated to be 0.62 for the oval-shaped cell (left) and 0.96 for the more rounded cell (right). The p-value used in the comparison of the roundness between the CTCs isolated between the different draws was calculated using the Wilcoxon rank-sum test. (DOCX) [file pone.0101777.s003.docx]

**Figure S3.**


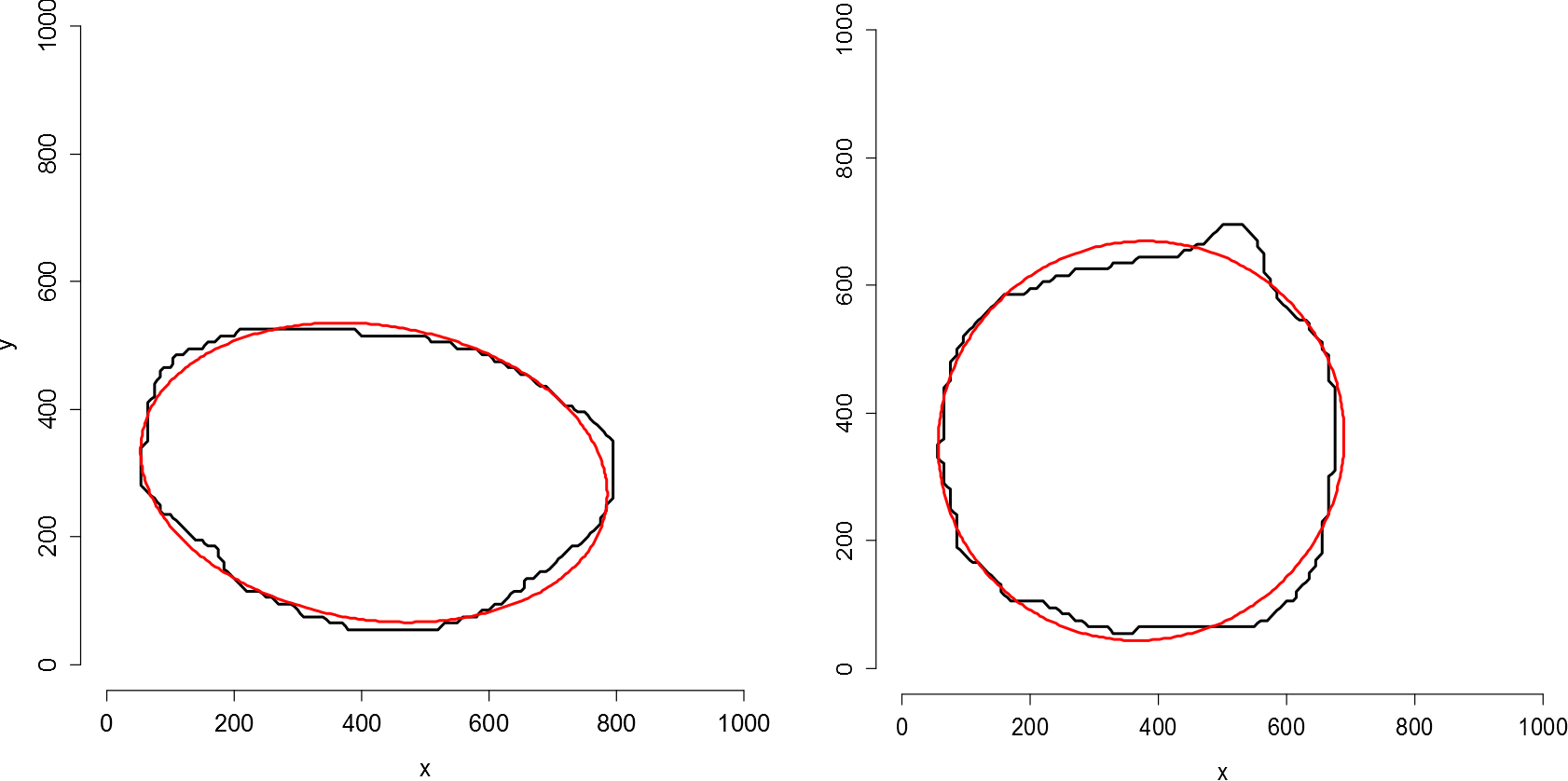


**Figure S3. Examples of cell roundness estimation**. The cell shape was analyzed by tracing the cell cytoplasm contour in the composite image of each CTC. The traced cell image was imported into R, and an ellipsis was fitted to the shape using a least squares fitting algorithm described by Halir and Flusser [1]. Black line represents the manually drawn cell outline, red line the fitted ellipse. The cell roundness is estimated as the fraction of the *de facto* cell area and the area of a circle with the radius set to the cell’s major axis. The cell roundness calculated to be 0.62 for the oval-shaped cell (left) and 0.96 for the more rounded cell (right). The p-value used in the comparison of the roundness between the CTCs isolated between the different draws was calculated using the Wilcoxon rank-sum test.

**Supplementary Reference**

1. Halir R, Flusser J (1998) Numerically stable direct least squares fitting of ellipses. Proceeding of International Conference in Central Europe on Computer Graphics, Visualization and Interactive Digital Media: 125-132.
