# Supplementary material for: Rapid Phenotypic and Genomic Change in Response to Therapeutic Pressure in Prostate Cancer Inferred by High Content Analysis of Single Circulating Tumor Cells
Source: PLoS One. 2014 Aug 1;9(8):e101777. doi: 10.1371/journal.pone.0101777 (PMC4118839; doi:10.1371/journal.pone.0101777)
Supplement: Table S1 — Summary of the different phenotypic and genotypic traits analyzed in the 41 individual cells profiled for copy number alterations. Concordance between AR phenotype-genotype was determined by comparison of the AR amplification status with the AR staining phenotype (Negative or Positive) for each individual cell. In red are cells that exhibited discordant AR phenotype-genotype. (DOCX) [file pone.0101777.s004.docx]

**Table S1.**

| **Cell Nomenclature** | **Draw** | **Cluster** | **AR gene status** | **AR protein Staining** | **AR signal intensity** | **CK signal intensity** | **Cell Roundness** |
| --- | --- | --- | --- | --- | --- | --- | --- |
| p33164.2.2.CAS0524 | 1 | B | No AR Amp | Negative | 2.54 | 67.22 | 0.925969 |
| p33164.2.4.CAS0520 | 1 | A | AR Amp | Positive | 41.14 | 172.33 | 0.845638 |
| p33164.2.17.CAS0522 | 1 | A | AR Amp | Positive | 84.68 | 100.14 | 0.927069 |
| p33164.1.5.CAS0498 | 1 | B | No AR Amp | Positive | 11.68 | 70.91 | 0.756361 |
| p33164.2.6.CAS0521 | 1 | A | AR Amp | Positive | 23.27 | 154.74 | 0.80859 |
| p33164.2.20.CAS0523 | 1 | A | AR Amp | Positive | 106.40 | 65.24 | 0.966375 |
| p33164.1.12.CAS0501 | 1 | A | AR Amp | Positive | 20.98 | 80.76 | 0.963558 |
| p33164.2.21.CAS0525 | 1 | A | AR Amp | Positive | 69.59 | 42.92 | 0.846964 |
| p33164.1.13.CAS0502 | 1 | A | AR Amp | Positive | 32.78 | 68.95 | 0.874024 |
| p33164.2.22.CAS0526 | 1 | A | AR Amp | Positive | 132.3 | 51.96 | 0.87912 |
| ARPred.1.2.CAS0568 | 2 | A | AR Amp | Positive | 11.00 | 114.09 | 0.806197 |
| ARPred.1.3.CAS0569 | 2 | A | AR Amp | Positive | 46.05 | 32.61 | 0.642296 |
| ARNred.1.2.CAS0589 | 2 | A | AR Amp | Negative | 1.59 | 160.01 | 0.893199 |
| p33164.5.2.CAS0533 | 2 | A | AR Amp | Positive | 84.30 | 42.84 | 0.737126 |
| p33164.5.4.CAS0534 | 2 | A | AR Amp | Positive | 92.47 | 69.28 | 0.729997 |
| p33164.5.6.CAS0535 | 2 | A | AR Amp | Positive | 47.80 | 125.17 | 0.370085 |
| ARPred.1.4.CAS0570 | 2 | A | AR Amp | Negative | 2.77 | 46.68 | 0.512653 |
| p33164.5.7.CAS0536 | 2 | A | AR Amp | Positive | 90.00 | 49.65 | 0.854095 |
| ARNred.1.5.CAS0592 | 2 | A | AR Amp | Positive | 9.11 | 95.68 | 0.920361 |
| ARNblue.1.1.CAS0595 | 3 | B | No AR Amp | Negative | 1.71 | 18.44 | 0.699269 |
| p33164.6.29.CAS0561 | 3 | B | No AR Amp | Negative | 5.98 | 44.98 | 0.793052 |
| p33164.6.15.CAS0559 | 3 | B | No AR Amp | Negative | -0.17 | 16.89 | 0.700298 |
| p33164.6.16.CAS0560 | 3 | B | No AR Amp | Negative | 2.97 | 33.33 | 0.795308 |
| p33164.6.32.CAS0562 | 3 | B | No AR Amp | Negative | 1.92 | 77.42 | 0.464112 |
| ARNblue.1.6.CAS0599 | 3 | B | No AR Amp | Negative | 3.82 | 98.88 | 0.886125 |
| p33164.6.34.CAS0563 | 3 | B | No AR Amp | Negative | 2.23 | 43.11 | 0.633107 |
| p33164.6.36.CAS0564 | 3 | B | No AR Amp | Negative | 0.33 | 14.63 | 0.804486 |
| p33164.6.37.CAS0565 | 3 | C | AR Amp | Negative | 0.92 | 34.28 | 0.614988 |
| ARNblue.1.5.CAS0598 | 3 | B | No AR Amp | Negative | 1.22 | 33.02 | 0.673803 |
| ARNblue.1.8.CAS0601 | 3 | B | No AR Amp | Negative | 1.25 | 22.36 | 0.560946 |
| ARNblue.1.7.CAS0600 | 3 | A | AR Amp (low) | Negative | 0.53 | 9.302 | 0.422198 |
| p33164.7.22.CAS0632 | 4 | A | AR Amp | Positive | 37.82 | 50.80 | 0.878598 |
| ARPblack.1.2.CAS0603 | 4 | C | AR Amp | Positive | 16.80 | 108.81 | 0.932083 |
| ARPblack.3.2.CAS0618 | 4 | C | AR Amp | Positive | 85.29 | 116.14 | 0.512847 |
| ARPblack.1.4.CAS0613 | 4 | C | AR Amp | Positive | 57.45 | 27.05 | 0.68 |
| p33164.7.27.CAS0634 | 4 | A | AR Amp | Positive | 9.11 | 37.54 | 0.936407 |
| p33164.7.28.CAS0636 | 4 | A | AR Amp | Positive | 19.09 | 27.47 | 0.932493 |
| p33164.7.30.CAS0638 | 4 | A | AR Amp | Negative | 0.65 | 19.72 | 0.903884 |
| ARPblack.3.3.CAS0619 | 4 | C | AR Amp | Positive | 38.64 | 59.20 | 0.901781 |
| p33164.7.37.CAS0642 | 4 | C | AR Amp | Positive | 38.76 | 16.79 | 0.858456 |
| ARblack.1.6.CAS0616 | 4 | C | AR Amp | Positive | 9.56 | 15.50 | 0.835043 |

**Table S1**. **Summary of the phenotypic and genotypic traits profiled in the 41 individual cells during treatment**. The concordance between AR phenotype-genotype was determined by comparison of the AR amplification status with the AR staining phenotype (Negative or Positive) for each individual cell. Cells that exhibited discordant AR phenotype-genotype were colored in red (cells with AR negative immunostaining in which AR was amplified or vice versa). To define AR positivity, a value of more than 6 standard deviations over the mean signal intensity (SDOM) observed in the leukocytes (background) was used as threshold. The values for the CK signal intensity reported as SDOM and cell roundness were also measured for each single cell.
